# Expression and role of oncogenic miRNA-224 in esophageal squamous cell carcinoma

**DOI:** 10.1186/s12885-015-1581-6

**Published:** 2015-08-06

**Authors:** Xiaoyan He, Zhimei Zhang, Ming Li, Shuo Li, Lihua Ren, Hong Zhu, Bin Xiao, Ruihua Shi

**Affiliations:** 1 Department of Gastroenterology, The First Affiliated Hospital of Nanjing Medical University, 300 Guangzhou Road, Nanjing, China; 2 Department of Gastroenterology, Dongyang People’s Hospital, 60 Wuningxi Road, Jinhua, China; 3 Department of Gastroenterology, The First People’s Hospital of Lianyungang, 182 Tongguanbei Road, Lianyungang, China; 4 Department of Gastroenterology, Friendship Hospital of Yangzhou, 440 Siwangting Road, Yangzhou, China; 5 Department of Gastroenterology, Zhangjiagang First People’s Hospital, 68 Jiyangxi Road, Suzhou, China; 6 Department of Gastroenterology, Zhongda Hospital, Southeast University, 87 Dingjiaqiao Road, Nanjing, China

## Abstract

**Background:**

Aberrant expression of miR-224 is associated with tumor development and progression. This study investigated the role of miR-224 in esophageal squamous cell carcinoma (ESCC) *ex vivo* and *in vitro*.

**Methods:**

A total of 103 esophageal intraepithelial neoplasia, ESCC tissue specimens, and their matched distant normal tissues were collected to test miR-224 expression using qRT-PCR analysis. Western blot was used to quantify the level of PH domain leucine-rich repeat protein phosphatase 1 (PHLPP1) and PHLPP2 in ESCC tissues. Cell viability, apoptosis, invasion, and colony formation assays were used to assess the altered phenotypes of esophageal cancer cell lines after miR-224 expression or inhibition. A luciferase reporter assay was used to confirm miR-224 binding to *PHLPP1* and *PHLPP2* mRNA.

**Results:**

miR-224 was significantly overexpressed in esophageal intraepithelial neoplasia and ESCC tissues, while the expression of PHLPP1 and PHLPP2 proteins, the target genes of miR-224, was downregulated in ESCC tissues. miR-224 expression was associated with advanced clinical TNM stage, pathologic grade, and the level of PHLPP1 and PHLPP2 proteins in ESCC tissues. Ectopic overexpression of miR-224 promoted proliferation, migration, and invasion, but suppressed apoptosis of ESCC cells. miR-224 was able to bind to the 3′ untranslated region (3′-UTR) of *PHLPP1* and *PHLPP2* mRNA to suppress their expression.

**Conclusions:**

The current study demonstrated that miR-224 acts as an oncogenic miRNA in ESCC, possibly by targeting PHLPP1 and PHLPP2.

**Electronic supplementary material:**

The online version of this article (doi:10.1186/s12885-015-1581-6) contains supplementary material, which is available to authorized users.

## Background

Esophageal cancer is a lethal disease with poor prognosis. A large percentage of patients with esophageal cancer are diagnosed at the advanced stages of disease [1, 2]. Histologically, esophageal cancer occurs in two major forms, esophageal squamous cell carcinoma (ESCC) and esophageal adenocarcinoma, each of which has distinct geographic patterns of incidence and risk factors [3, 4]. Stretching from northern Iran through the central Asian republics to North-Central China, ESCC is the predominant histological subtype, accounting for 90 % of the total esophageal cancer cases [1]. To date, surgery is the only cure option to treat esophageal cancer patients, but this only applies to limited numbers of patients due to their inoperable disease; thus, the overall five-year survival rate for the patients is 14 to 20 % [2]. Although multiple genetic and epigenetic alterations have been detected in ESCC [5, 6], the precise pathogenesis of ESCC remains to be discovered. Molecular markers for early diagnosis and prediction of prognosis or treatment responses are quite limited [4]. Thus, further studies on the targeted prevention and early detection of esophageal cancer could help to limit the lethality of this disease.

microRNA (miRNA) is a class of small-regulatory non-coding RNA molecules with 18 to 22 nucleotides long. It can post-transcriptionally regulate gene expression through pairing with the 3′-untranslated region (UTR) of the targeted messenger RNAs (mRNA) and control translation or induce degradation of target gene. miRNA plays an important role in basic physiological processes in cells, such as cell growth, differentiation, apoptosis, energy metabolism, and immune response [7]. Previous studies demonstrated that aberrant miRNA expression can act as either a tumor suppressor or oncogene [8, 9]. A number of miRNA expression profiling studies have been conducted in ESCC, and the expression of miRNAs, including miR-21, miR-25, and miR-223 have been shown to be altered. These miRNAs could be further evaluated as biomarkers for association with ESCC progression and clinical outcome [9, 10]. Other studies showed that dysregulation of miR-145 and miR-195 was able to modulate ESCC cell viability, proliferation, invasion, and metastasis [8, 11]. In our unpublished study, we identified several differentially expressed miRNAs in ESCC tissues, compared to paired distant normal tissues, using the Agilent microarray and found several high expressed miRNAs, including miR-244. Indeed, miR-224 has been reported to be dysregulated in various human malignancies and can potentially affect many cancer-related cellular processes, including gene transcription, proliferation, differentiation, and cell death [12, 13]. Bioinformatical studies have shown that miR-224 may target PH domain leucine-rich repeat protein phosphatase (PHLPP)-1 and PHLPP2, both of which function as a tumor suppressor by blocking Akt signaling. Down-regulation of PHLPP1 and PHLPP2 proteins has been found in a variety of malignant tumors, including colorectal cancer [14], prostate cancer [15], and chronic lymphocytic leukemia (CLL) [16].

In this study, we first assessed miR-244 expression in esophageal intraepithelial neoplasia biopsies, ESCC tissues, and their matched distant normal tissues, then associated the expression pattern with clinicopathological features from ESCC patients. We also examined the effects of miR-224 expression or inhibition on regulation of ESCC cell viability and mobility and investigated the underlying molecular events in esophageal cancer cells *in vitro*.

## Methods

### Tissue samples

In this study, we collected 103 cases of esophageal patients from General Surgery Department and Digestive Disease Department, The First Affiliated Hospital of Nanjing Medical University (Jiangsu, China), between January 2013 and December 2013. Patients who had received radiotherapy, chemotherapy, or other esophageal surgery before esophagectomy were excluded. Specifically, we obtained 10 pairs of low-grade intra-epithelial neoplasia (LG-IEN) biopsies and their matched distant normal tissues (5 cm away from the lesion), 30 pairs of HG-IEN biopsies and their matched distant normal tissues, and 63 pairs of ESCC tissues and their matched normal esophageal tissues (5 cm away from tumor lesion according to NCCN guideline of esophageal cancer) for this study. Patients with a postoperative pathological result including both IEN and ESCC were included into ESCC group. And only those paitients who had IEN only were included in the IEN group. All tissue specimens were immediately snap-frozen in liquid nitrogen and then stored at −80 °C. This study was approved by the Ethics Committee of the First Affiliated Hospital of Nanjing Medical University, and a written informed consent was obtained from each patient.

### RNA isolation and quantitative reverse-transcriptase-polymerase chain reaction (qRT-PCR)

Total cellular RNA was isolated from tissue specimens or cell lines using the Trizol reagent (TaKaRa, Dalian, China), according to the manufacturer’s instructions. For qRT-PCR, 2 μg each of these RNA samples were reversely transcribed into cDNA using PrimeScript RT Master Mix (TaKaRa), and PCR amplification was performed at an initial 95 °C for 5 min and then 40 cycles of 95 °C for 20 s, 60 °C for 20 s, and 70 °C for 10 s in the StepOnePlus™ PCR system (ABI, USA) using SYBR Premix Ex Taq (Takara), according to the manufacturer’s procedures. The qRT-PCR miRNA Primer Set (one RT primer and a pair of qPCR primers for each set) specific for miR-224 was designed and synthesized by RiboBio (Guangzhou, China). U6 small nuclear RNA was used to normalize miR-224 expression. All qRT-PCR amplification was performed in triplicate and repeated at least once. The relative miR-224 expression levels after normalization to U6 small nuclear RNA were calculated using 2^-[(Ct of miR-224) – (Ct of U6)]^. The increasing fold of miR-224 were expressed relative to the matched normal tissues and calculated using 2^-ΔΔCT^(ΔΔCT = ΔCT_IEN/ESCC_-ΔCT_Normal_). All data after logarithm transition (y = LnX) were applied for the statistical analysis.

### Cell lines and culture

Human ESCC cell line TE13 and Eca109 were obtained from Shanghai Institute of Biochemistry and Cell Biology (Shanghai, China) and cultured in Rosewell Park Memorial Institute (RPMI)-1640 medium with 10 % fetal bovine serum (FBS; Invitrogen, Carlsbad, CA, USA) in a humidified incubator with 5.0 % CO_2_ at 37 °C. To avoid possible effects on gene expression, antibiotic was not used in cell culture.

### Transient gene transfection into ESCC cell lines

hsa-miR-224 mimic, miR-224 negative control (NC), miRNA-224 inhibitor, and miRNA-224 inhibitor negative control (INC) were purchased from Genepharma (Shanghai, China). hsa-miR-224 mimic and NC were incomplete complementary double stranded, while miRNA-224 inhibitor and INC were single stranded. All oligonucleotide sequences are listed in Table 1. ESCC cells were seeded and grown overnight and next day, these RNA oligonucleotides were transiently transfected into cells using Lipofectamine 2000 (Invitrogen), according to the manufacturer’s protocol. After 24 or 48 h incubation, the cells were harvested and subjected to different experiments.

**Table 1:** Oligonucleotide sequences

Name	Oligonucleotide sequences	
miR-224 mimics	sense	5′-CAAGUCACUAGUGGUUCCGUU-3′
	antisense	5′-CGGAACCACUAGUGACUUGUU-3′
NC	sense	5′-UUCUCCGAACGUGUCACGUTT-3′
	antisense	5′-ACGUGACACGUUCGGAGAATT-3′
miR-224 inhibitors	single strand	5′-AACGGAACCACUAGUGACUUG-3′
INC	single strand	5′-CAGUACUUUUGUGUAGUACAA-3′

### Cell viability assay

After transfection with aforementioned RNA oligonucleotides, TE13 and Eca109 cells were seeded in 96-well plates at a density of 4000 cells/well and cultured for 24 to 96 h. At the end of each experiment, Cell Counting Kit-8 (Dojindo, Shanghai, China) reagents were added into each well and the plates were incubated for 1.5 h, and the optical density (OD) was then measured at 450 nm using NanoDrop 2000 (Thermo, USA). The experiments were performed in 5 replicates and repeated three times.

### Colony formation assay

Forty-eight h after transfection with RNA oligonucleotides, cells were trypsinized and plated on 6-well plates at a density of 300 cells/well and cultured for 10 days. The colonies were then fixed with methanol for 15 min and stained with 0.4 % crystal violet at the room temperature for 30 min. The number of colonies, which were defined as > 50 cells/colony, were counted. The experiments were performed in triplicate and repeated three times.

### Flow cytometric apoptosis assay

Forty-eight hours after transfection with RNA oligonucleotides, cells were harvested through trypsinization and washed two times with phosphate buffered saline (PBS). Apoptosis was assessed using a FITC Annexin V Apoptosis Kit (Becton Dickinson, Franklin Lakes, NJ, USA), according to the manufacturer’s instructions. Cells were then analyzed using a flow cytometer (Becton Dickinson) within 1 h of staining using the FL1-H (FITC) and FL2-H (PI) lines.

### Tumor cell migration and invasion assay

Tumor cell migration and invasion were assayed using 24-well Transwell plates with 8 mm pore size (Millipore, Bedford, MA, USA). For migration assay, 48 h after transfection with RNA oligonucleotides, ESCC cells were trypsinized, washed, resuspended in 2 % FBS-containing medium, and added to the upper chamber, while 15 % FBS-containing medium was added to the bottom chambers. The Transwell chambers were incubated at 37 °C for 48 h and non-migrating cells were removed by a cotton swab, while the migrated cells were fixed, stained with crystal violet, and counted for ten random fields/chamber. The experiments were conducted in triplicate and repeated three times. Tumor cell invasion assay was the same as the migration assay, except that the membranes were coated with Matrigel (BD) and the Transwell plates were fixed and stained 72 h after the invasion assay.

### Luciferase assay

Using online tools, including miRBase, TargetScan, and miRDB, we found that PHLPP1 and PHLPP2 are potentially targeted by miR-224. The miR-224 binding site is localized between 518 to 524 bp of the *PHLPP1* 3′-UTR (a total of 1002 bp long) or between 3562 to 3568 bp of the *PHLPP2* 3′-UTR (3941 bp long). The sequence of PHLPP1 pGL3-3′-UTR-wt (5′-AUAUGGAGACUAACUCCUAGGAGUUGCUUUACUCUGUCAGGUGACUUAAGUCACUGGGAUUCACUAAUUUUCUCUGAGAGAACAGCUG-3′), pGL3-3′-UTR-mut (5′-AUAUGGAGACUAACUCCUAGGAGUUGCUUUACUCUGUCAGUUAUGAUCAGUCACUGGGAUUCACUAAUUUUCUCUGAGAGAACAGCUG-3′), PHLPP2 pGL3-3′-UTR-wt (5′-CCGACUCCCAAUCAUGAAGGCAAGUUAAUCUUUCCAGUUAGUGACUUUUGCCCCAUAGUUGGGGUAAGCACUUVVUAGAUUGAAAA-3′), and pGL3-3′-UTR-mut (5′-CCGACUCCCAAUCAUGAAGGCAAGUUAAUCUUUCCAGUUAUACUGUAUUGCCCCAUAGUUGGGGUAAGCACUUCCUAGAUUGAGAAAA-3′) were generated by Invitrogen-China. Eca109 and TE13 cells were then co-transfected with pGL3-3′-UTR and miR-224 mimic or inhibitor, or their matched miR-negative control. After 24 h transfection, Firefly and Renilla luciferase activity was measured using the Dual Luciferase Assay kit (Promega, Madison, WI, USA), according to the manufacturer’s instructions. The data were normalized against the activity of the Renilla luciferase. The experiments were performed in triplicate and repeated three times.

### Protein extraction and Western blot

Tissue specimens and cells were homogenized, and the total cellular protein was extracted using the RIPA Lysis Buffer (Beyotime, Jiangsu, China), according to the manufacturer’s instructions. Protein concentration was measured by the BCA method (Beyotime). Western blot was performed using anti-phospho-AKT, anti-AKT, anti-GAPDH (Cell Signaling Technology, Danvers, MA, USA), anti-PHLPP1, and anti-PHLPP2 (Abcam, Cambridge, MA, USA) antibodies. The relative levels of protein expression were quantified by densitometric scanning (Image J Software, NIH, Bethesda, MD, USA), and the relative gray values of protein levels were calculated based on the band intensity of protein of interest divided by the band intensity of loading control.

### Immunofluorescence

Seventy-two h after gene transfection, Eca109 cells were fixed in 4 % paraformaldehyde for 15 min at the room temperature, washed three times in PBS, and incubated for 60 min at the room temperature in a blocking solution (Beyotime). The primary antibody (anti-PHLPP1 and anti-PHLPP2 from Abcam) in the blocking reagent was added and incubated overnight at 4 °C. The next day, the cells were washed with PBS for three times, and the fluorescent probe conjugated secondary antibody (Jackson ImmunoResearch, USA) was added to the cells. The cells were further incubated for 60 min at the room temperature and then washed three times in PBS. Cell nucleus was counterstained with DAPI (Beyotime). Photos were then taken using a Nikon Fluorescence microscope.

### Statistical analysis

The data were summarized as mean ± S.D. and analyzed using Student’ *t*-test, Chi-square test, mono factor analysis of variance, and the correlation analysis with SPSS 17.0 software (SPSS, Chicago, IL, USA). A *p*-value less than 0.05 was considered to be statistically significant.

## Results

### Overexpression of miR-224 is in esophageal intraepithelial neoplasia and ESCC tissue specimens

**Fig. 1:**
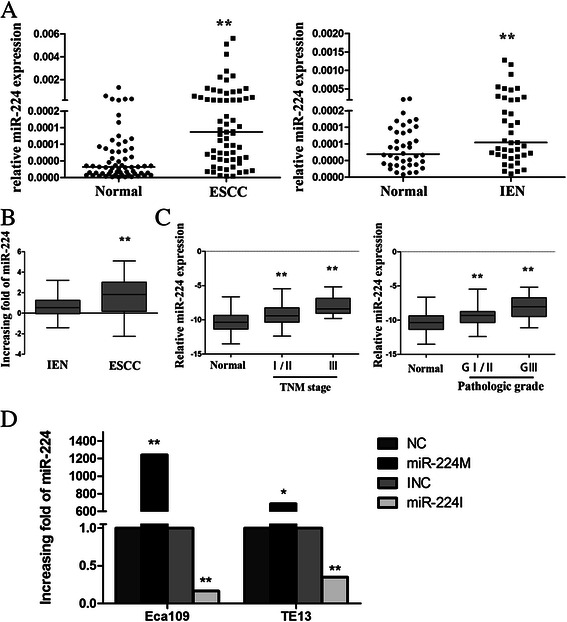
Overexpression of miR-224 in esophageal intraepithelial neoplasia and ESCC tissues. **a** qRT-PCR. miR-224 expression was assessed in 40 pairs of IEN (intraepithelial neoplasia) biopsies and their matched adjacent normal tissues and 63 pairs of ESCC tissues and their matched normal esophageal tissues. Each point represents the mean of three independent experiments. The horizontal lines represent the median in each group. **b** Comparison of miR-224 expression in tissues obtained from ESCC and IEN patients. The horizontal lines represent the mean in each group. **c** Comparison of mean miR-224 expression stratified by TNM and pathologic classification. The horizontal lines represent the mean in each group. **d** qRT-PCR. Expression of miR-224 in esophageal cancer Eca109 and TE13 cell lines after transfected with miR-224 mimics or inhibitors. *p < 0.05 and **p < 0.01

miR-224 was significantly overexpressed in esophageal intraepithelial neoplasia and ESCC tissue samples compared to that of the matched distant normal tissues (*p* < 0.05, Fig. 1a; Additional file 1: Figure S1 and Additional file 2: Figure S2). Specifically, qRT-PCR data showed that expression of miR-224 was increased for more than two folds in 70 % of ESCC patients compared to normal mucosae (Additional file 1: Figure S1). Moreover, miR-224 expression was significantly different between ESCC and IEN (*p* < 0.001, Fig. 1b). To further investigate clinicopathological significance of miR-224 expression in ESCC patients, we chose the mean of fold increase of miR-224 expression in ESCC samples as the cut-off point of low and high expression of miR-224. We found that miR-224 expression was associated with ESCC pathologic grade (*p* < 0.01, Fig. 1c and Table 2) and TNM stage (*p* < 0.01, Fig. 1c and Table 2), but not with other clinicopathological data from the patients (Table 2).

**Table 2:** Association of miR-224 expression with clinicopathological features from ESCC patients

Characteristics	miR-224 expression		*P* value^a^
	High	Low	
Age (years)			
≥60	22	15	0.658
<60	14	12	
Gender			
Male	28	17	0.198
Female	8	10	
Tumor localization^b^			
Upper third	3	5	0.442
Middle third	17	10	
Lower third	16	12	
Tumor length (cm)			
<3	15	12	0.881
3–5	17	13	
>5	4	2	
Pathologic grade			
GI	5	8	0.035
GII	10	12	
GIII	21	7	
TNM			
I	5	6	0.029
II	16	18	
III	15	3	

^a^Chi-square test

^b^Classified according to the AJCC, 7th edition, staging criteria for esophageal cancer

### Ectopic expression of miR-224 changed phenotypes of ESCC cell *in vitro*

Next, we altered miR-224 expression in esophageal cancer cell lines. As shown in Fig. 1d, transfection with miR-224 mimics or inhibitor significantly up- or down-regulated the level of miR-224 expression in Eca109 and TE13 cells compared to the control oligonucleotides-transfected cells.

**Fig. 2:**
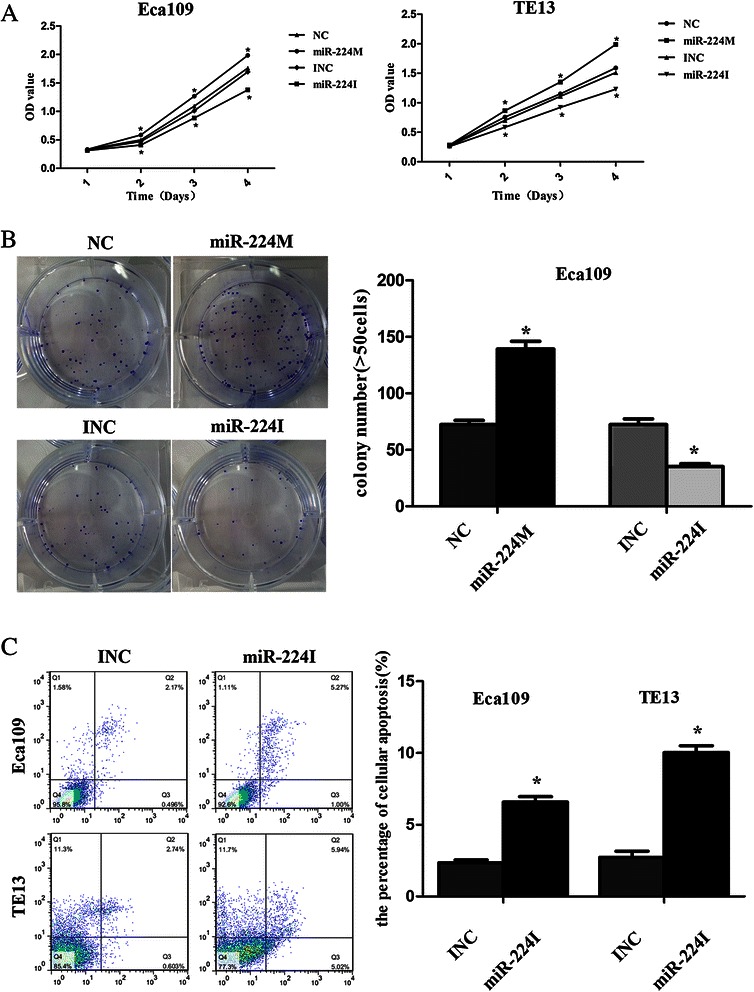
Ectopic miR-224 expression changed the phenotypes of ESCC cell *in vitro*. **a** Cell viability CCK-8 assay. The effects of miR-224 on ESCC cell proliferation ability. **b** Colony formation assay. The effects of miR-224 on Eca109 cell line colony formation ability. **c** Flow cytometric apoptosis assay. miR-224I increased apoptosis of ESCC cell lines compared to the negative control. Apoptotic cells (%) = Q2 + Q3. Error bars represent the mean ± SD of three independent experiments. OD, optical density; miR-224 M, miR-224 mimic; miR-224I, miR-224 inhibitor; NC, negative control; *p < 0.05 and **p < 0.01

We then assessed the phenotypes of Eca109 and TE13 cell lines. The CCK-8 assay revealed that overexpression of miR-224 significantly increased viability of Eca109 and TE13 cell lines compared to negative control (NC)-transfected cells, whereas transfection of anti-miR-224 reduced tumor cell viability (*p* < 0.05, Fig. 2a). Moreover, tumor cell colony formation assay showed that miR-224 mimic increased the colony-forming efficiency of Eca109 cells, whereas anti-miR-224 suppressed the colony-forming efficiency of Eca109 cells (*p* < 0.05, Fig. 2b). In addition, the flow cytometry assay showed that anti-miR-224 increased apoptosis of both ESCC cell lines compared to the negative control (*p* < 0.05, Fig. 2c).

**Fig. 3:**
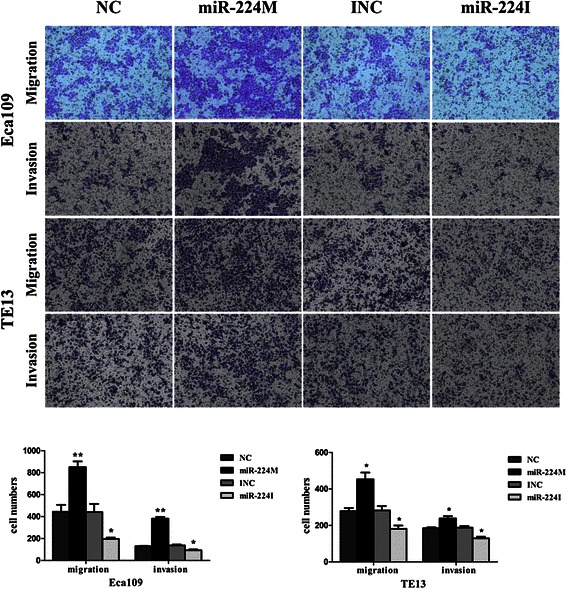
Ectopic miR-224 expression altered the ability of ESCC cell migration and invasion. Esophageal cancer Eca109 and TE13 cell lines were grown and transiently transfected with miR-224 mimics or inhibitor, and then subjected to migration and invasion assays. The data showed that overexpression of miR-224 significantly promoted migration and invasion of Eca109 and TE13 cells, whereas anti-miR-224 significantly suppressed migration and invasion. Error bars represent the mean ± SD of three independent experiments. miR-224 M, miR-224 mimic; miR-224I, miR-224 inhibitor; NC, negative control; *p < 0.05 and **p < 0.01

Furthermore, as shown in Fig. 3, overexpression of miR-224 significantly promoted migration and invasion of Eca109 and TE13 cell lines, whereas anti-miR-224 suppressed migration and invasion of these two cell lines compared to the negative control.

### miR-224 directly targets tumor suppressor gene PHLPP1 and PHLPP2 in ESCC cells

**Fig. 4:**
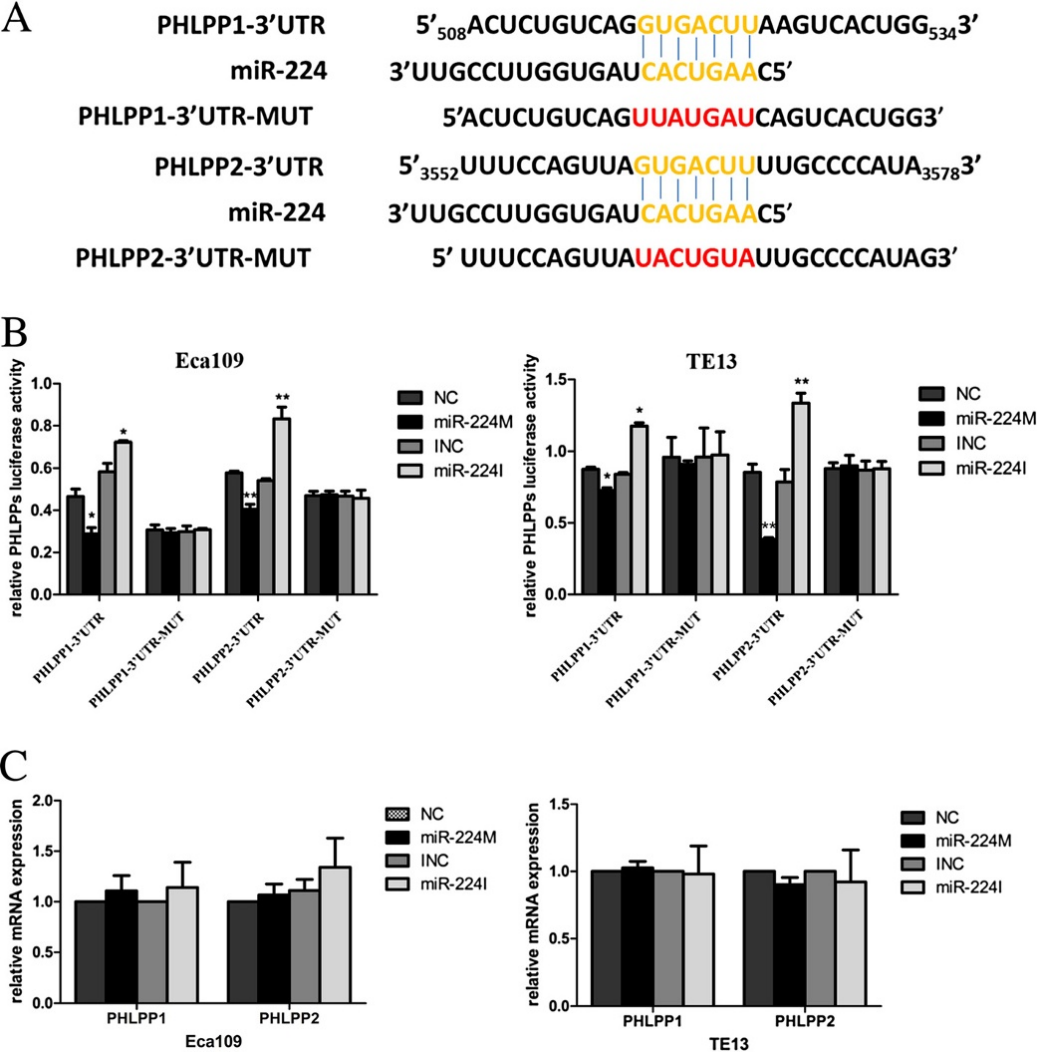
PHLPP1 and PHLPP2 as target genes of miR-224 in ESCC cells. **a** Predicted miR-224 target sequences in *PHLPP1* and *PHLPP2* 3′-UTRs. The mutated nucleotides in PHLPP1 and PHLPP2 3′-UTRs used for our experiments are highlighted in red. **b** Luciferase assay. Interaction of miR-224 with 3′-UTR of *PHLPP1* and *PHLPP2* was confirmed by the luciferase assay in Eca109 and TE13 cells. **c** qRT-PCR. The data showed that miR-224 did not affect expression of PHLPP1 and PHLPP2 mRNA in Eca109 and TE13 cells (*p* > 0.05). Error bars represent the mean ± SD of three independent experiments. miR-224 M, miR-224 mimic; miR-224I, miR-224 inhibitor; NC, negative control; *p < 0.05 and **p < 0.01

**Fig. 5:**
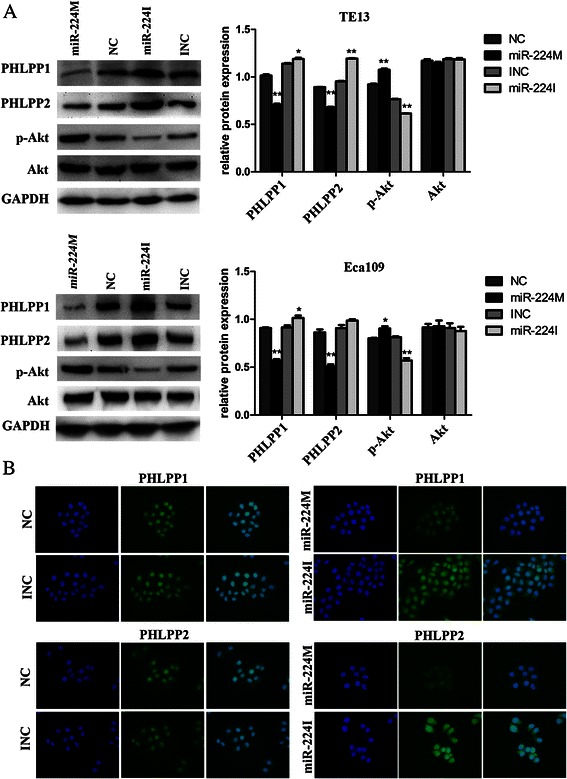
Ectopic miR-224 expression affected expression of PHLPP1 and PHLPP2 proteins and changed the AKT signaling in ESCC cells. **a** Western blot. Esophageal cancer Eca109 and TE13 cell lines were grown and transiently transfected with miR-224 mimics and inhibitor, and then subjected to Western blot analysis. **b** Immunofluorescence analysis of PHLPP1 and PHLPP2 expression in transfected Eca109 cell line. From the left to right, the first panel is DAPI staining, the second is PHLPP1/2 staining, and the third one is overlap. Error bars represent the mean ± SD of three independent experiments. miR-224 M, miR-224 mimic; miR-224I, miR-224 inhibitor; NC, negative control; *p < 0.05 and **p < 0.01

To predict miR-224 targets, we utilized Bioinformatical algorithms of miRBase, TargetScan, and miRDB and showed that miR-224 could bind to the 518–524 bp and 3562–3568 bp, 3′-UTR region of *PHLPP1* and *PHLPP2*, respectively (Fig. 4a). To confirm this prediction, we constructed luciferase reporter vectors and performed the luciferase reporter assay. The data showed that miR-224 mimic significantly reduced both PHLPP1 and PHLPP2 reporter gene luciferase activity compared to the controls. In contrast, miR-224 inhibitor significantly promoted both PHLPP1 and PHLPP2 reporter gene luciferase activity (*p* < 0.05, Fig. 4b). Mutations of the potential miR-224-binding sites of PHLPP1 and PHLPP2 3′-UTRs prevented the ability of miR-224 mimics and inhibitor to alter luciferase activity, suggesting that miR-224 is able to target the predicted PHLPP1 and PHLPP2 binding site. Indeed, qRT-PCR, immunofluorescence, and Western blotting data showed no change in the levels of PHLPP1 or PHLPP2 mRNA in miR-224-transfected cells compared to the controls (*p* > 0.05, Fig. 4c), whereas levels of their proteins were dramatically down-regulated in miR-224-overexpressing cells and up-regulated after inhibition of miR-224 expression in esophageal cancer cell lines (*p* < 0.05, Fig. 5). These data indicated that miR-224 affects translation of PHLPP mRNA.

### Ectopic expression of miR-224 affected the Akt signaling in ESCC cells

To explore the possible underlying molecular mechanisms of miR-224 action in ESCC cell lines, we analyzed the downstream signals of PHLPP1 and PHLPP2. As shown in Fig. 5a, phosphorylation levels of AKT protein was increased in miR-224-overexpressing ESCC cells, whereas anti-miR-224 suppressed the levels of phosphorylated AKT protein compared to the negative control.

### Down-regulated PHLPP1 and PHLPP2 expression associated with miR-224 expression in ESCC tissues

**Fig. 6:**
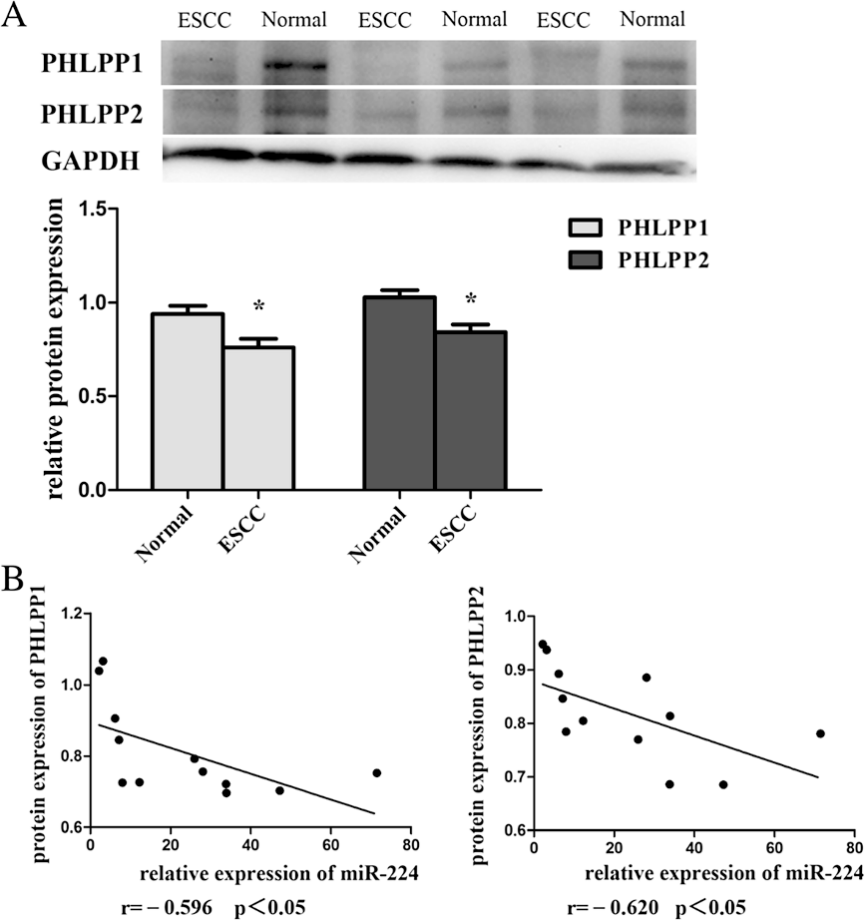
Down-regulated of PHLPP1 and PHLPP2 expression associated with miR-224 in ESCC. **a** ESCC and their matched normal esophageal tissue specimens from 12 patients were subjected to Western blot analysis. The relative gray values of protein were calculated as band intensity of protein of PHLPPs/band intensity of GAPDH. Error bars represent the mean ± SD of three independent experiments. **b** Correlation analysis of miR-224 expression and PHLPPs protein levels in ESCC tissues. *p < 0.05 and **p < 0.01c

The level of PHLPP1 and PHLPP2 protein was significantly reduced in 12 cases of ESCC tissue samples compared to that of the matched distant normal tissues (*p* < 0.05, Fig. 6a and Additional file 3: Figure S3). Correlation analysis showed that expression of PHLPP1 and PHLPP2 proteins was associated with miR-224 expression in ESCC tissues (the correlation coefficient was −0.596 and −0.620, respectively; *p* < 0.05, Fig. 6b).

## Discussion

In the current study, we first assessed miR-244 expression in esophageal IEN biopsies, ESCC tissues, and their matched distant normal tissues, and associated the expression pattern with clinicopathological features from ESCC patients. We also investigated the effects of miR-224 expression or inhibition on regulation of ESCC cell viability and invasion capacity, and determined the underlying molecular mechanisms in esophageal cancer cells *in vitro*. We found that miR-224 was up-regulated in esophageal IEN and ESCC tissue specimens compared to that of the matched distant normal tissues, and miR-224 expression was associated with aggressive characteristics of IEN and ESCC. Furthermore, ectopic overexpression of miR-224 promoted proliferation, migration, and invasion, but suppressed apoptosis of ESCC cells *in vitro*. miR-224 was able to bind to *PHLPP1* and *PHLPP2* 3′-UTR and suppressed their expression and may subsequently induced activity of the AKT signaling, while the levels of PHLPP1 and PHLPP2 proteins were associated with miR-224 expression in ESCC tissues. Our current data indicates that miR-224 is an oncogenic miRNA and targets expression of PHLPP1 and PHLPP2 proteins in ESCC.

Nevertheless, it should be noted that miR-224 can be up-regulated or down-regulated in different types or subtypes of cancer. For example, miR-224 expression was frequently up-regulated in hepatocellular carcinoma [17], colorectal cancer [18], medulloblastoma [19], thyroid cancer [20], pancreatic ductal adenocarcinoma [21], renal cancer [22], cervical cancer [23], and glioma [24]. In contrast, down-regulation of miR-224 has been observed in prostate cancer [25], ovarian cancer [26], giant cell tumor [27], and oral cancer [28]. These data suggest that miR-224 can function as an oncogene or tumor suppressor through regulating the expression of different target genes. For example, in hepatocellular carcinoma, ectopic overexpression of miR-224 significantly down-regulated HOXD10 expression and promoted cell migration and invasion [12], whereas inhibition of miR-224 expression enhanced cell migration and invasion of prostate cancer cells through direct regulation of oncogenic TPD52 [29]. In support of the tumor-promoting role of miR-224 in ESCC, our current data showed that miR-224 was up-regulated in esophageal IEN and ESCC tissue specimens compared to that of the matched distant normal tissues, and miR-224 expression was associated with aggressive characteristics of IEN and ESCC. These data further confirmed that miR-224 can function as an oncogene by regulating the expression of PHLPP1 and PHLPP2 proteins.

Furthermore, our current data also showed that ectopic overexpression of miR-224 promoted proliferation, migration, and invasion, but suppressed apoptosis of ESCC cells *in vitro*, which is consistent with the data on HCC [12]. Importantly, our current study further confirmed that PHLPP1 and PHLPP2 are two target genes of miR-224. Both of PHLPP1 and PHLPP2 proteins contain each of a PH domain, leucine-rich repeat, phosphatase domain, and PDZ-binding motif [30]. As tumor-suppressor phosphatases, PHLPP1 and PHLPP2 proteins act to suppress cell survival pathways. They can directly dephosphorylate a conserved regulatory site (termed the hydrophobic motif) of Akt protein, protein kinase C, and S6 kinase. In turn, these phosphatases block signaling of these pro-survival kinases, and promote apoptosis and suppress tumor growth [30, 31]. A number of studies showed altered expression of PHLPP1 and PHLPP2 and their functions in various malignancies [32, 33]. Indeed, a previous study showed expression and association between miR-224 and PHLPPs in colorectal cancer [13]. Another study reported by Cai et al. [34] suggested that PHLPP2 was a *bona fide* target gene of miR-205 and associated the up-regulation of miR-205 with cell growth and vascularization. Our current data further demonstrated that the loss of PHLPP1 and PHLPP2 expression occurred in ESCC tissue samples and their expression was associated with miR-224 expression in ESCC tissues. These data revealed the mechanisms responsible for PHLPP expression, because to date, little is known regarding regulation of PHLPP expression in different human cells [35].

However, our current study is limited to proof-of-principle and further investigations are needed to understand the role of miR-224 in esophageal cancer and clarify how miR-224 regulates expression of PHLPP1 and PHLPP2 proteins during esophageal cancer compared to other cancer pathogenesis.

## Conclusions

In summary, our current study showed that miR-224 up-regulation could promote ESCC development, possibly through inhibiting PHLPP1 and PHLPP2 expression, indicating that miR-224 has oncogenic activity in ESCC. These findings may not only increase our understanding of ESCC development and progression, but may also help us to develop a novel therapeutic strategy for ESCC.

## Additional files

Additional file 1: Figure S1. Expression of miR-224 in 63 pairs of ESCC and their matched normal esophageal tissue specimens. The data were quantified by using ΔΔCT = ΔCT_ESCC_-ΔCT_Normal_. (TIFF 235 kb)
Additional file 2: Figure S2. Expression of miR-224 in 40 pairs of IEN (intraepithelial neoplasia) biopsies and their matched adjacent normal tissues. The data were quantified by using ΔΔCT = ΔCT_IEN_-ΔCT_Normal_. (TIFF 486 kb)
Additional file 3: Figure S3. Expression of PHLPP1 and PHLPP2 in 12 pairs of ESCC and their matched normal esophageal tissue specimens was detected by Western blot. The relative gray values of protein were calculated as band intensity of protein of PHLPPs/band intensity of GAPDH. (TIFF 720 kb)
