# Supplementary figures and images for: Expression and role of oncogenic miRNA-224 in esophageal squamous cell carcinoma
Source: BMC Cancer. 2015 Aug 6;15:575. doi: 10.1186/s12885-015-1581-6 (PMC4545858; doi:10.1186/s12885-015-1581-6)

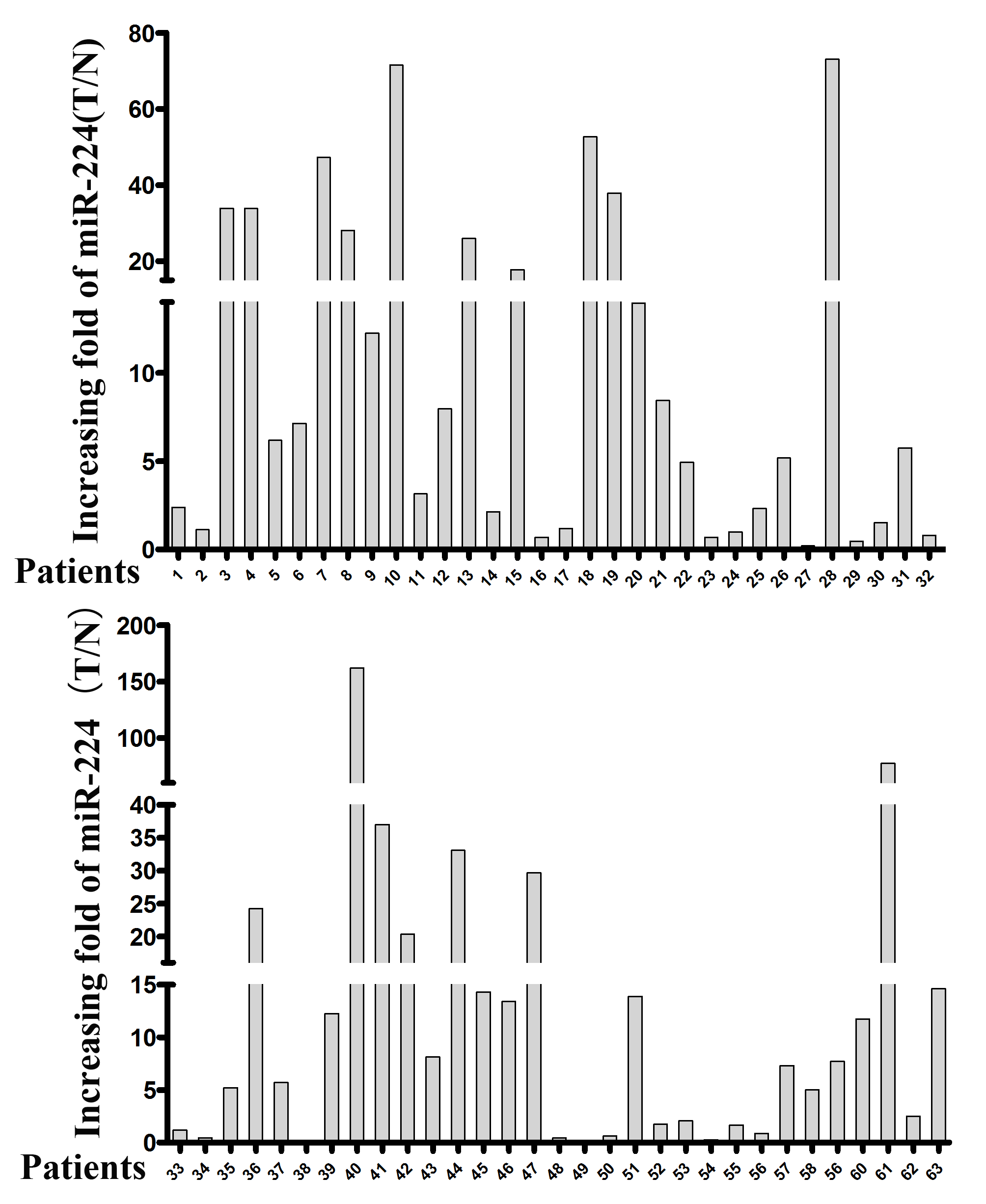

Supplement: Additional file 1: Figure S1. — Expression of miR-224 in 63 pairs of ESCC and their matched normal esophageal tissue specimens. The data were quantified by using ΔΔCT = ΔCTESCC-ΔCTNormal. (TIFF 235 kb) [file 12885_2015_1581_MOESM1_ESM.tiff]

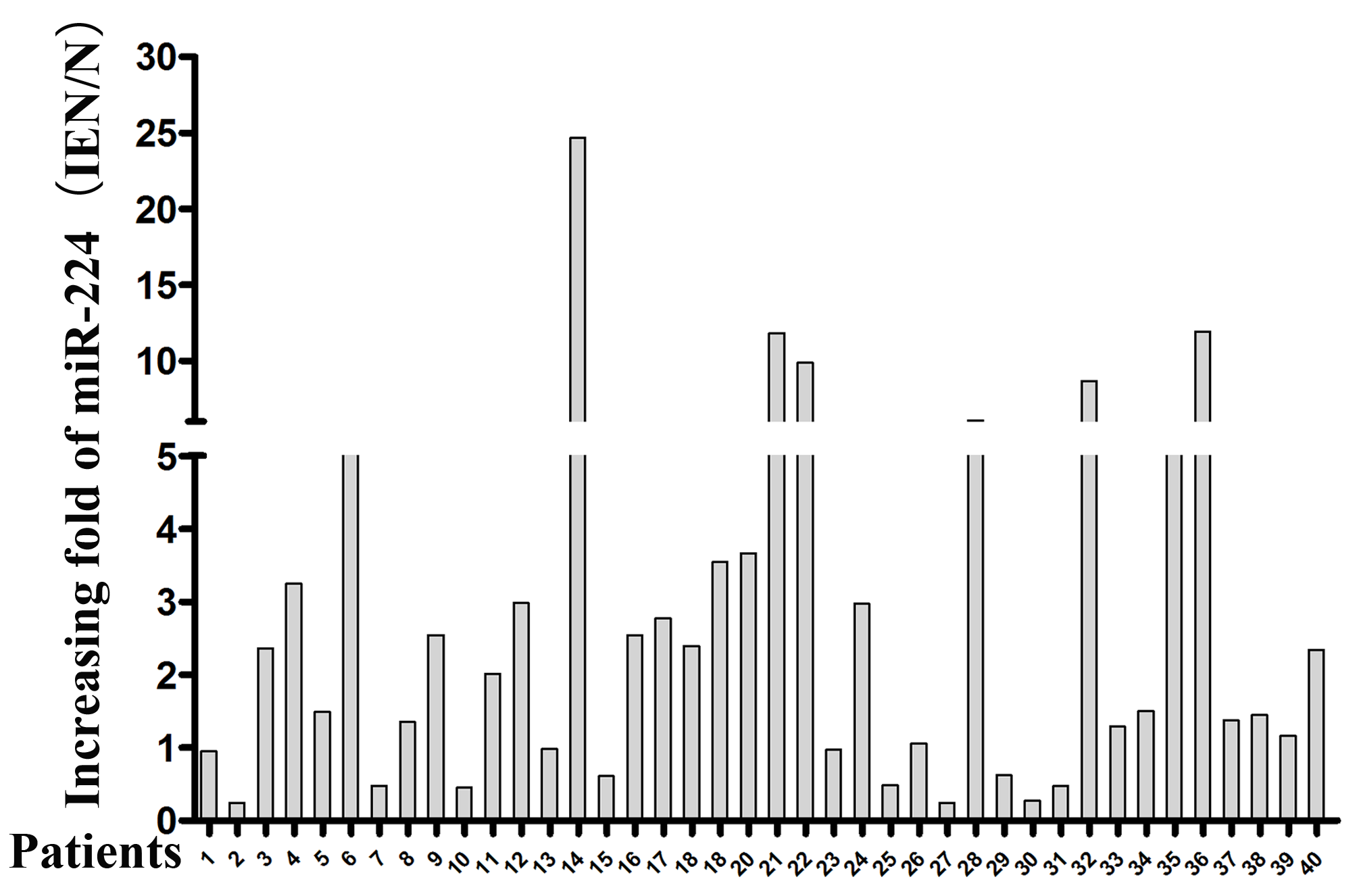

Supplement: Additional file 2: Figure S2. — Expression of miR-224 in 40 pairs of IEN (intraepithelial neoplasia) biopsies and their matched adjacent normal tissues. The data were quantified by using ΔΔCT = ΔCTIEN-ΔCTNormal. (TIFF 486 kb) [file 12885_2015_1581_MOESM2_ESM.tiff]

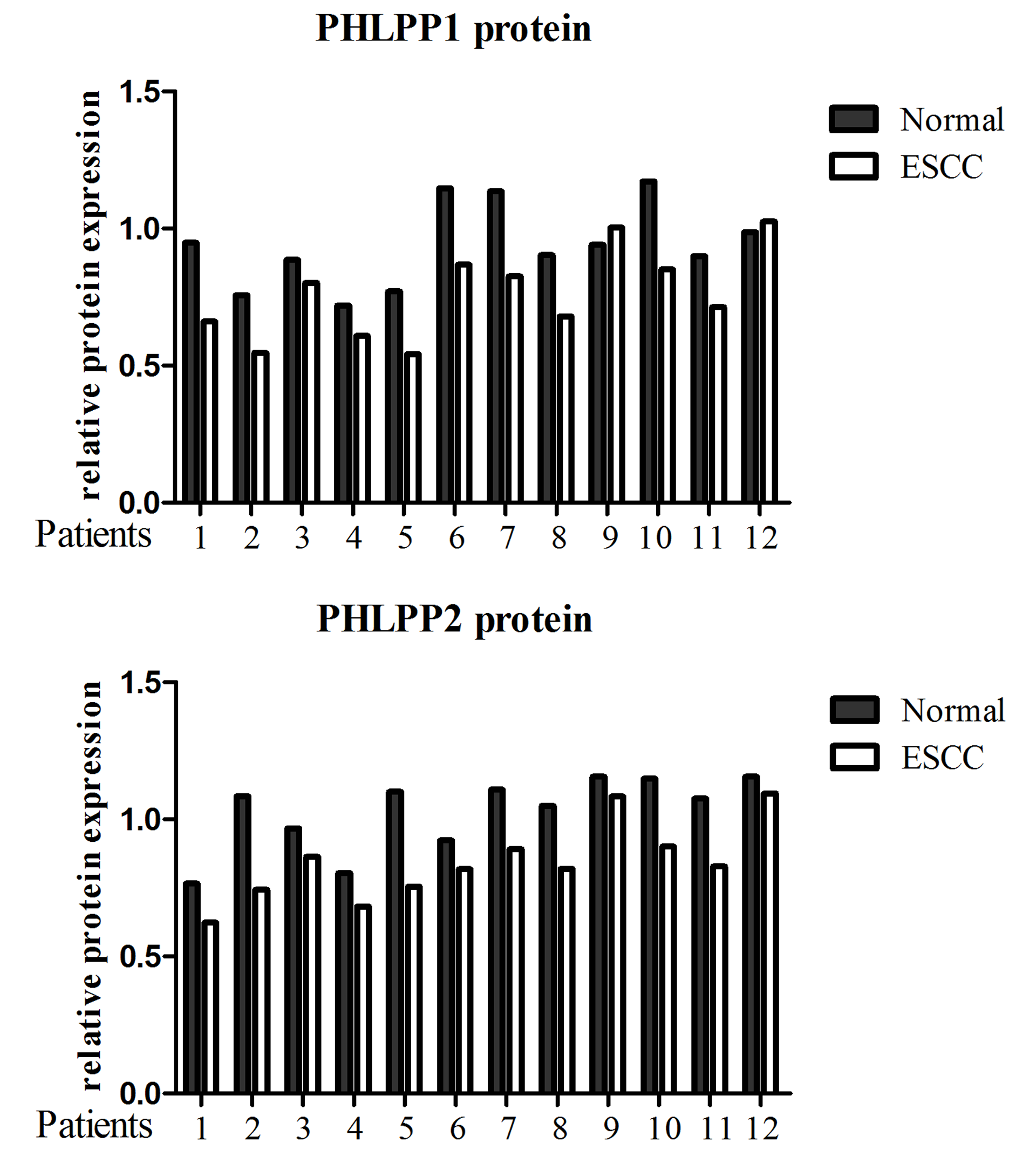

Supplement: Additional file 3: Figure S3. — Expression of PHLPP1 and PHLPP2 in 12 pairs of ESCC and their matched normal esophageal tissue specimens was detected by Western blot. The relative gray values of protein were calculated as band intensity of protein of PHLPPs/band intensity of GAPDH. (TIFF 720 kb) [file 12885_2015_1581_MOESM3_ESM.tiff]
